# Ipsilateral transfer of motor skill from upper to lower limb in healthy adults: a randomized controlled trial

**DOI:** 10.3389/fnhum.2025.1645986

**Published:** 2025-11-04

**Authors:** Orit Elion, Zvi Kozol, Moshe Einat, Silvi Frenkel-Toledo

**Affiliations:** ^1^Department of Physical Therapy, School of Health Sciences, Ariel University, Ariel, Israel; ^2^Department of Electrical and Electronic Engineering, Ariel University, Ariel, Israel; ^3^Department of Neurological Rehabilitation, Loewenstein Medical Rehabilitation Center, Ra’anana, Israel

**Keywords:** ipsilateral transfer, motor performance, upper limb, lower limb, cognition

## Abstract

Intermanual transfer refers to the improvement of motor skill in an untrained contralateral limb following unilateral limb practice. However, it remains uncertain whether motor skill in the lower limb (LL) can improve as a result of practice with the unilateral upper limb (UL). Forty-five healthy participants were randomly allocated to one of three groups: (1) UL group, which practiced reaching movement (RM) sequences with the non-dominant left upper limb; or (2) switches observation (SO) group, which observed the same RM sequences; or (3) nature observation (NO) group, which observed nature movies. RM performance with the LL was assessed before, immediately after, and 24 h post-intervention. Response time of RM sequences was faster in the UL group than the NO group in the posttest. Response time improved significantly in the posttest and retest compared to the pretest in all groups, but it improved significantly in the retest compared to the posttest only in the NO group. The percentage of fails to reach within 1 s decreased across all time points in all groups. The combination of practice of the RM sequence with the UL and the cognitive engagement during RM sequence observation contributes to ipsilateral transfer from the UL to the LL.

## 1 Introduction

Skilled performance becomes more specific with increased practice (Rozanov et al., 2010; Keetch et al., 2008; Sosnik et al., 2004; Korman et al., 2003). However, at early phase of motor learning, some practiced skills may be transferred to different skills or to other effectors (e.g., the contralateral limb) (Schmidt and Young, 1987; Karni et al., 1998). The transfer to other effectors occurs because the acquired memory includes components that are effector-independent (Kumar et al., 2020). The specificity of a learned task entails increased dependence on the physical and contextual parameters of the training experience and emerges following extensive training (Rozanov et al., 2010; Keetch et al., 2008; Sosnik et al., 2004; Korman et al., 2003; Karni et al., 1995, 1998; Hikosaka et al., 1999). Once the learned skill has become specific in the long-term memory, it is difficult to apply knowledge of the learned task condition to a novel task condition, i.e., transfer of the gains from the learned task to a novel task is less possible. The amount of transfer or specificity depends on the level of central nervous system representation (Korman et al., 2003; Karni et al., 1998; Farthing and Zehr, 2014) such that lower-level representation of the task in the central nervous system indicates that the task is less abstract and more specific, and less transfer is possible.

Intermanual transfer has been extensively investigated in the domains of strength training (Magnus et al., 2010; Farthing and Zehr, 2014; Green and Gabriel, 2018; Manca et al., 2017; Munn et al., 2005) and motor skill acquisition (Kumar et al., 2020; Aune et al., 2017; Müssgens and Ullén, 2015; Pan and Van Gemert, 2013; Berner and Hoffman, 2009; Japikse et al., 2003; Kumar and Mandal, 2005) in healthy individuals. Unilateral strength training has been shown to produce significant strength increases in the untrained contralateral limb, with gains of up to 29% in the untrained side (Farthing and Zehr, 2014). Regarding motor skill transfer, improvements such as faster reaction times of finger sequence were transferred from the trained effector to the contralateral untrained effector (Berner and Hoffman, 2009; Japikse et al., 2003). Additionally, the speed component of a star tracing task (Kumar and Mandal, 2005) and movement trajectories of a reaching adaptation task (Kumar et al., 2020) were intermanually transferred.

In contrast to the evidence on intermanual transfer of the UL (Farthing and Zehr, 2014; Green and Gabriel, 2018; Manca et al., 2017; Munn et al., 2005; Aune et al., 2017; Müssgens and Ullén, 2015; Pan and Van Gemert, 2013; Berner and Hoffman, 2009; Japikse et al., 2003; Kumar and Mandal, 2005), research on ipsilateral transfer remains relatively limited (Magdi et al., 2021; Ben Othman et al., 2018; Hansen et al., 2001). Some studies have suggested the possibility of strength transfer from the LL to the UL. For example, an increase in the one-repetition maximum of the ipsilateral biceps brachii was found after a 10-weeks leg press resistance training program targeting the LL (Ben Othman et al., 2018). Additionally, training the UL biceps muscle immediately followed by leg press exercises was found to be more effective in enhancing UL isometric biceps strength than biceps training alone (Hansen et al., 2001).

To the best of our knowledge, only some studies investigated ipsilateral transfer of motor skills (Sherman et al., 2024; Debarnot et al., 2024; Saeedpour-Parizi et al., 2022) and prism-induced visuomotor adaptation (Savin and Morton, 2008). For example, Sherman et al. (2024) investigated if a unilateral UL motor skill, consisting of sequential reaching movement, can improve following practice of that skill with the LL whereas Debarnot et al. (2024) and Saeedpour-Parizi et al. (2022) investigated the transfer of bilateral interlimb sequential motor learning. Sherman et al. (2024) found that reaching response time improved in the group that practiced sequential reaching movements with the LL more than in the control groups (who either observed a sequence of light switches or watched nature films) in the posttest. Additionally, the LL group showed more improvement than the latter control group in the retest conducted 24 h later. Debarnot et al. (2024) found that reciprocal transfer gains in performance were observed regardless of the UL or LL practiced. Greater transfer gains in performance were observed at the start of the transfer from the LLs to the ULs (44%) but these gains dropped to 5% after practice with the transfer effectors. In contrast, the transfer from the ULs to LLs initially resulted in smaller gains (15%), but these gains persisted and remained significant at 9% following practice with the transfer effectors.

This study is the first attempt to determine whether ipsilateral transfer of motor skill occurs from the unilateral UL to the LL [whereas Sherman et al. (2024), investigated transfer in the opposite direction and found unilateral transfer from the LL to the UL, and other studies examined bilateral limb motor skill transfer (Debarnot et al., 2024; Saeedpour-Parizi et al., 2022)]. This aim is theoretically important for elucidating motor learning principles. It also has potential practical implications, as goal-directed movements of the lower limb (LL)–and not only of the UL–such as donning footwear or trousers, are fundamental components of daily activities. Specifically, we investigated whether practicing reaching movement (RM) sequences with the UL toward illuminating switches can be transferred to the LL in healthy adults. The process of sequence learning consists of two distinct components: first, acquiring the order of elements within the sequence, and second, the ability to execute the sequence by integrating these elements into a cohesive skilled action (Farthing et al., 2009). Real-world motor tasks inherently include cognitive as well as motor components (Krakauer et al., 2019). We compared the practice of RM sequences to merely observing the same sequences of light switches to assess the contribution of the cognitive component (related to sequence memory) versus the combined cognitive and motor components in the ipsilateral transfer of RM sequences. We hypothesized that practicing RM sequences with the UL would improve the performance of RM sequences with the ipsilateral LL compared to merely observing the same sequences of the illuminating switches or observing nature movies.

## 2 Materials and methods

### 2.1 Study design

This was a single-blind, parallel, randomized, controlled study. Data were collected in a brain and motor behavior laboratory based at Ariel University, Israel. Subjects were randomly assigned with a 1:1:1 ratio, using a random number generator in WINPEPI, to one of three groups: (1) practice of RM sequence with the UL toward illuminating switches (UL group); (2) observation of sequence of illuminating switches [Switches Observation (SO) group]; and (3) observation of nature movies [Nature Observation (NO) group]. All participants were blinded to group allocation. Research assistants who administered the intervention and measured the outcomes received allocation information via coded email from the researcher SFT. Blinding of group allocation was maintained during the data analysis. The trial was retrospectively registered at the ClinicalTrials.gov registry on 01/03/2023 with trial registration number NCT05748769. The protocol is available on the following website: https://register.clinicaltrials.gov/prs/app/action/SelectProtocol?sid=S000CY74&selectaction=Edit&uid=U0005AKF&ts=2&cx=-9dosv4. All methods were performed in accordance with the relevant guidelines and regulations.

### 2.2 Participants

**FIGURE 1:**
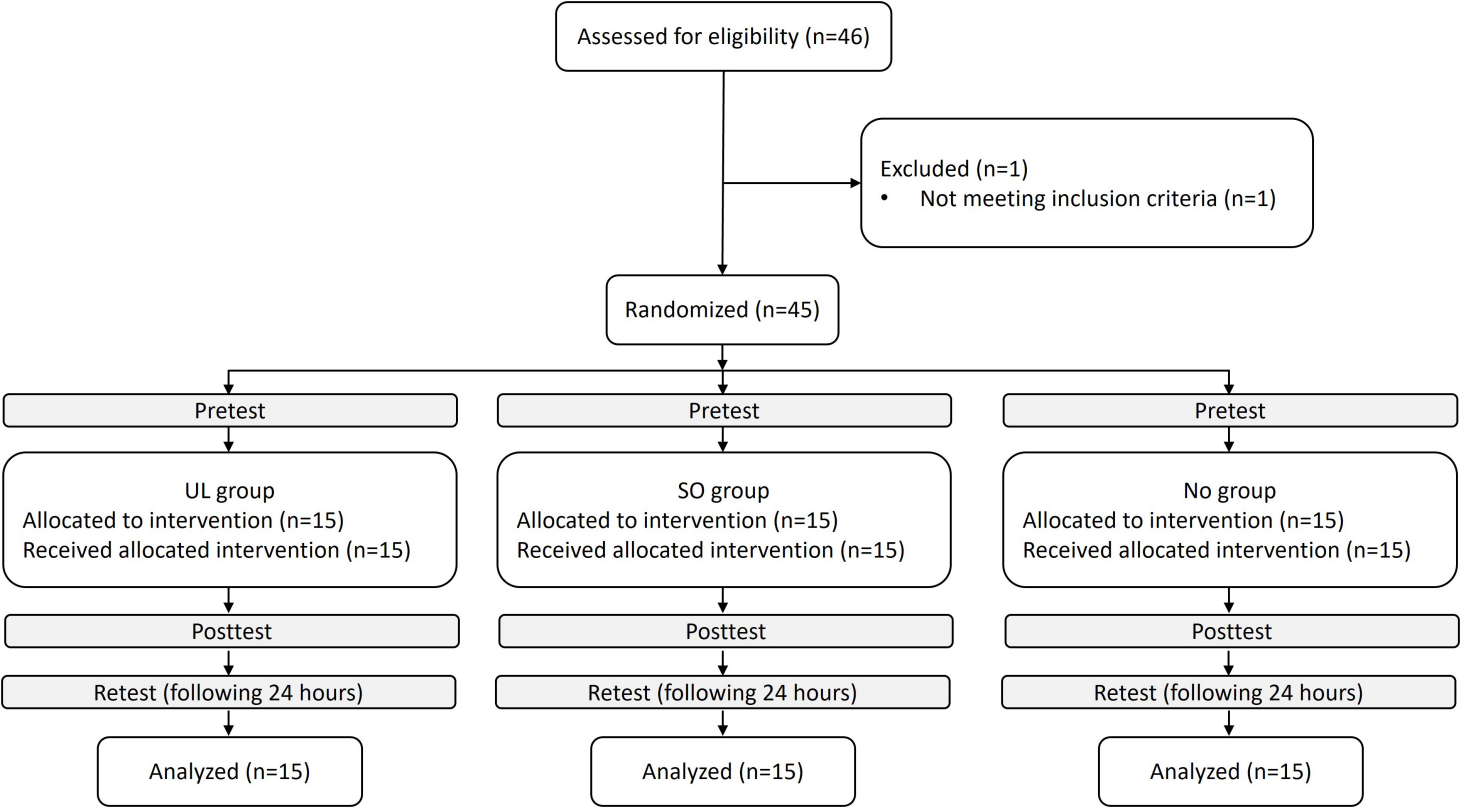
Trial flowchart. UL group, upper limb group that practiced the reaching movement sequence with the UL toward light switches; SO group, switches observation group that observed the sequence of light switches; NO group, nature observation group that observed nature films.

The sample size for this study was determined through a power analysis using G*Power version 3.1.9.7. The analysis indicated that a total of 45 participants (15 per group) would be necessary to detect a significant interaction, assuming an effect size f of 0.25 and a power of 90%. A total of 45 individuals (23 women; aged 24 ± 2 years) participated in the study between January 2022 and September 2022. The inclusion criteria required participants to be between the ages of 20 and 35, right-hand dominant, and self-reported as healthy. Exclusion criteria included any musculoskeletal or neurological deficits that could interfere with task performance, particularly UL and LL reaching tasks. The study was approved by the Ethics Committee of Ariel University (approval number: AU-HEA-OE-20210610). Written informed consent was obtained from all participants involved in the study. The Consolidated Standards of Reporting Trials (CONSORT) recommendations (CONSORT Checklist) are followed in our study; a CONSORT flow diagram is shown in Figure 1.

### 2.3 Motor task

Participants completed two sessions. The first session consisted of familiarization with the motor task, a pretest, a single-session intervention (based on group randomization), and a posttest immediately following the intervention. The second session included a retest conducted 24 h after the intervention. Familiarization practice and tests were performed with the LL, and the single session intervention (for the UL group) was performed with the UL. At the beginning of each session, participants reported the previous night’s sleep duration and rated their sleep quality (good/not good).

Recording apparatus used in tests (pretest, posttest, and retest): A custom-made testing device was set up on a rectangular table with a smooth laminated tabletop of 105 cm × 80 cm and adjustable height. Five switch-led units of 5 cm × 8 cm × 5 cm, each composed of a large push-button switch and a red light-emitting diode (LED), were attached to the tabletop in a 38-cm radius half circle, successively numbered from 1 to 5. Activation of a specific unit LED was a cue for the subject to reach toward that unit and press the push-button switch. Reaching toward the switch of an activated unit deactivated it, and the response time between the activated and deactivated LED was recorded. A detailed description of the task and the apparatus is provided in previous studies (Sherman et al., 2024; Frenkel-Toledo et al., 2020).

**FIGURE 2:**
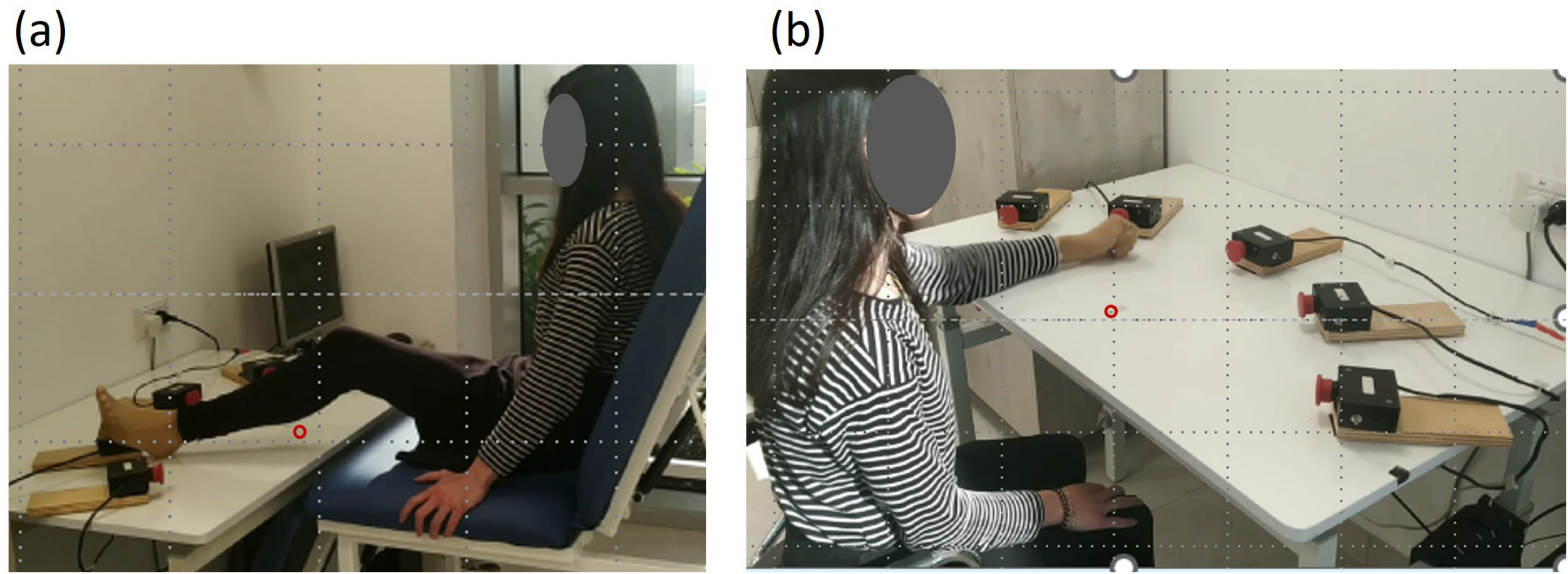
General setup. Five switch–LED units (5 × 8 × 5 cm) were arranged in a semicircle (38-cm radius) and numbered 1–5. Each unit included a push-button switch with a red light-emitting diode. The starting position is marked by a red circle. **(a)** Performance with the lower limb. **(b)** Performance with the upper limb.

To assess leg performance, participants sat on a custom-designed plinth with a solid back support positioned in front of the apparatus, which was set at the same height as the tabletop, allowing them to perform the RM sequence with their LL. In the starting position, the heel was placed at the edge of the table in front of the center of switch 3, with the left heel touching switch 3 and the knee flexed at 30° (Figure 2a).

Initially, participants completed a familiarization practice consisting of 30 randomized RM sequences. This involved reaching toward the activated unit with their left LL, touching the unit-related switch as quickly as possible, and returning the leg to the starting position before the next unit was activated. During the pretest, posttest, and retest, participants performed RMs with their left leg toward units activated in the sequence 1-4-3-5-4-2, with an activation duration and delay of 1 s. Participants were instructed to reach from the starting position to the illuminated switch as quickly and accurately as possible, press the switch, and return to the starting position, ensuring that their heel remained in contact with the table. They were not informed of the sequence.

During all three tests (pretest, posttest, retest), participants completed three blocks, with each block consisting of five sequences, resulting in a total of 15 sequences (90 RMs/trials overall). If a participant failed to reach the activated unit and touch the switch within 1 s, the trial was considered a “fail” and was excluded from the average response time. Participants rested for 30 s after each block. The primary outcome measure was the average response time of the RMs across all targets (ms), and the secondary outcome measure was the percentage of failed trials, calculated as (number of fails/30 trials) × 100. Improved motor performance was indicated by shorter response times and fewer failures.

### 2.4 Procedure of single session intervention

A 16-min single-session intervention was carried out in the UL, SO, and NO groups. During the intervention, participants’ initial testing position involved sitting on a chair with firm back support, with their hips and knees bent at a 90° angle, positioned in front of the testing apparatus. For the UL group, the starting position required participants to place their left fist on the edge of the table in front of their chest (parallel to the center of switch 3), allowing them to reach and touch switch 3 with the third metacarpal of their left hand. The UL group was instructed to reach with their left UL from the starting position as quickly and accurately as possible toward the illuminated switch, press it, and return to the starting position, ensuring that their fist remained in contact with the table. They were not informed of the sequence order. Participants performed RMs toward the units activated in the same sequence as the test 1-4-3-5-4-2, with a 1-s activation duration and delay. The practice session consisted of 16 blocks, each with 5 sequences (30 RMs), and participants rested for 30 s after each block (Figure 2b). The SO group was instructed to observe the illuminating switches without moving. The participants observed the RM sequence 1-4-3-5-4-2 with the same 1 s activation duration and delay, along with a 30 s pause after each block. The NO group was instructed to observe a video clip without moving. The video consisted of a 16-min nature film, with cycles of 1-min viewing followed by a 30-s pause, aligning with the timing of the RMs performed by the UL and SO groups.

Additional supporting experiment: To investigate the gains in performance of the trained UL and particularly the consolidation effects of the UL practice, we recruited additional 15 healthy subjects who performed a single session intervention of 16 blocks of RM sequences with the left UL. The procedures of familiarization, tests and intervention were identical to those described above (Sections “2.3 Motor task, 2.4 Procedure of single session intervention”) but were performed with the left UL.

### 2.5 Statistical analysis

Age and sex were compared between groups (UL, SO, NO) using Kruskal–Wallis (as age was not normally distributed) and chi-squared tests, respectively. Nocturnal sleep duration and sleep quality were compared across groups using Kruskal–Wallis (as sleep hours were not normally distributed) and chi-squared tests, respectively. Normal distribution was found for LL response time and not for percent of LL fails. Therefore, for the latter outcome measure, we used a log transform of the original value + 1 (adding the value of 1 is related to the fact that some subjects had zero failures) (the original values are presented for clarity). Differences between groups in the pretest, regarding each outcome measure (of the LL), were investigated using one-way ANOVA with Bonferroni correction for multiple comparisons. The effects of practice and time on the outcome measures were investigated using a mixed-design ANOVA with time (pretest, posttest, retest) as the within-subject factor and group (UL, SO, NO) as the between-subject factor with Bonferroni correction for multiple comparisons. For the additional supporting experiment, the effect of time on the outcome measures (of the UL) was investigated using a RM ANOVA with time (pretest, posttest, retest) as the within-subject factor. All tests were performed using SPSS (version 26.0) with initial significance levels of *p* < 0.05.

## 3 Results

Forty-six participants completed the pre-enrollment screening evaluation. Of those, one did not meet the inclusion criteria. Age (median [interquartile range]) (UL group: 26.0 [24–26] years; SO group: 26.0 [25–27] years; NO group: 26.0 [24–27] years) and sex (UL group: eight women; SO group: seven women; NO group: eight women) did not differ between groups (*p* > 0.590, for all). Nocturnal sleep duration (First session: UL group: 7 [5–7] h; SO group: 6 [6–7] h; NO group: 6 [6–7] h. Second session: UL group: 6 [6–7] h; SO group: 7 [6–7] h; NO group: 7 [6–8] h) and sleep quality [First session (number of participants reporting “good” sleep): UL group: 13; SO group: 13; NO group: 12. Second session: UL group: 14; SO group: 14; NO group: 15] did not differ between groups, in each session (*p* > 0.295, for all). Individual data are displayed in Supplementary Table 1.

### 3.1 Motor sequence learning task

Mean values of response time (s) and percent of fails by group and time are shown in Table 1. Response time and percent of fails did not show significant differences between groups in the pretest (*p* = 0.667, *p* = 0.715, respectively).

**TABLE 1:** Means, standard deviations and confidence intervals of response time and percent of fails for groups in time points.

Variable	UL (*n* = 15)			SO (*n* = 15)			NO (*n* = 15)		
	Pretest	Posttest	Retest	Pretest	Posttest	Retest	Pretest	Posttest	Retest
Response time (ms)	652.95 ± 59.28	432.09 ± 143.56	433.30 ± 149.15	657.68 ± 95.10	502.89 ± 144.74	460.57 ± 137.08	633.70 ± 72.24	582.55 ± 89.73	528.98 ± 107.29
	[620.12–685.78]	[352.59–511.59]	[350.71–515.90]	[605.01–710.35]	[422.74–583.05]	[384.66–536.49]	[593.69–673.71]	[532.86–632.24]	[469.56–588.39]
Fails (%)	6.44 ± 4.66	3.19 ± 3.66	2.22 ± 2.66	8.15 ± 6.01	2.59 ± 2.40	2.00 ± 1.53	7.63 ± 8.82	5.70 ± 6.31	3.26 ± 4.02
	[3.86–9.03]	[1.16–5.2]	[0.75–3.70]	[4.82–11.48]	[1.26–3.92]	[1.16–2.85]	[2.75–12.51]	[2.21–9.20]	[1.04–5.48]

UL, upper limb group which practiced reaching movement sequence with the UL toward illuminating switches; SO, switches observation group which observed the sequence of illuminating switches; NO, nature observation group which observed nature movies. ms, milliseconds.

**FIGURE 3:**
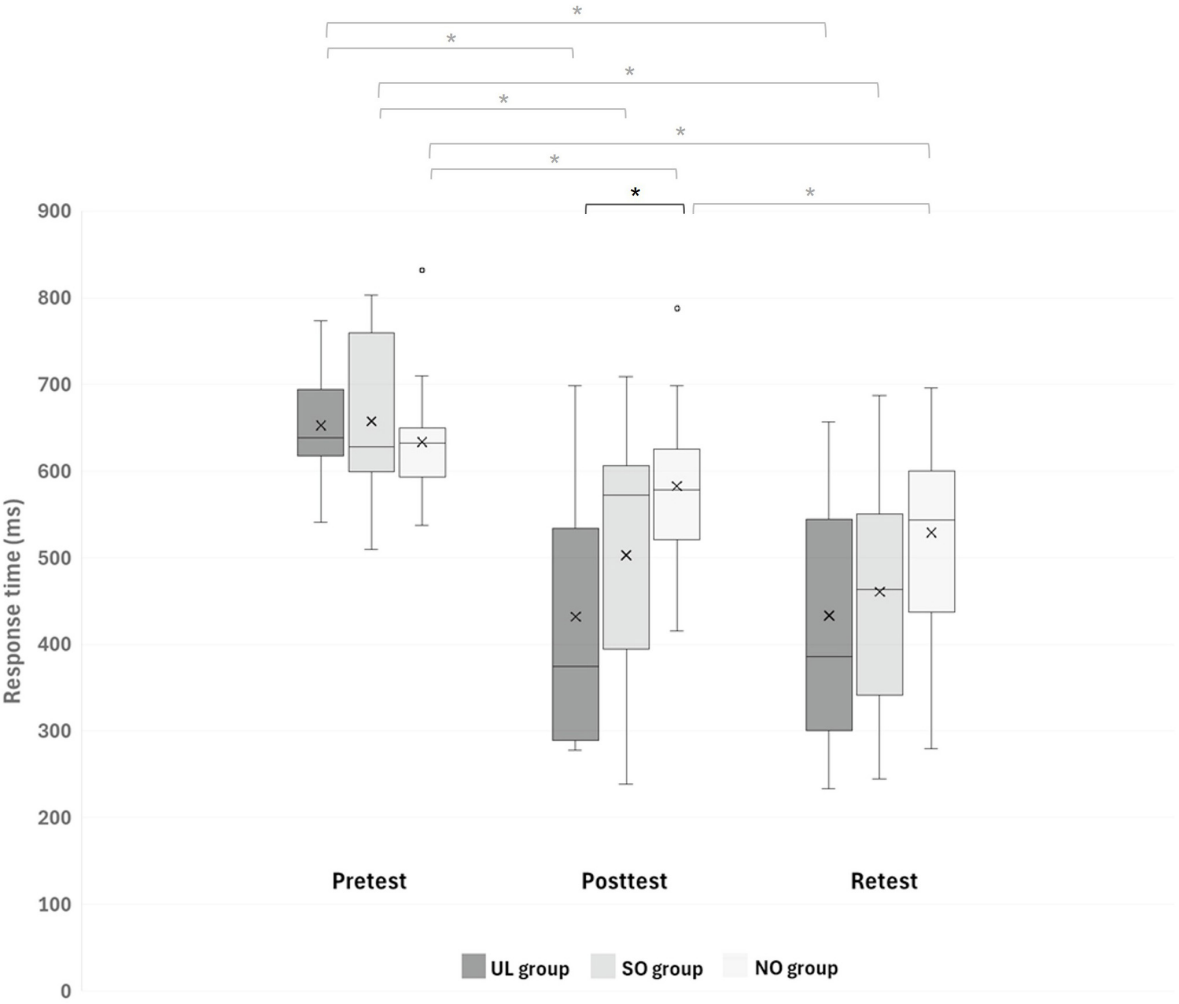
Response time (ms) of reaching movements (RMs) during all sequences in each group at different time points. Asterisks denote a significant difference (pBonferroni < 0.05). Black asterisk indicates differences between groups at a specific time point, while gray asterisks indicate differences between time points within each group. UL group, upper limb group that practiced the RM sequence with the LL toward light switches; SO group, switches observation group that observed the sequence of light switches; NO group, nature observation group that observed nature films.

Effects on response time (ms):

A main effect of Time (*F*(2,84) = 91.441; *p* < 0.001; partial η^2^ = 0.69; observed power = 1.00) showed that, overall, response time was shorter in the posttest (505.85 ± 75.94 ms) and retest (474.29 ± 135.56 ms) than in the pretest (648.11 ± 140.17 ms; pBonferroni < 0.001, for both) and in the retest than in the posttest (pBonferroni = 0.003). This effect was, however, modulated by Group, as was shown by the interaction of Group x Time (*F*(4,84) = 7.028, pBonferroni < 0.001; partial η^2^ = 0.25; observed power = 0.99). Figure 3 presents between-group differences at each time point (pretest, posttest, retest) and within-group differences across time. Only in the posttest, response time differed between groups (*F*(2,44) = 5.140; *p* = 0.010) such that it was significantly shorter in the UL group (432.09 ± 143.56 ms) than in the NO group (582.55 ± 89.73 ms; pBonferroni = 0.008). In addition, only in the NO group, response time decreased significantly in the retest (528.98 ± 107.29 ms) compared to the posttest (582.55 ± 89.73 ms) (pBonferonni = 0.004), whereas in each group, response time decreased significantly in the posttest and retest compared to the pretest (pBonferroni < 0.001, for all). No other significant effects were observed.

Effects on percent of fails (%):

A main effect of Time (*F*(2,84) = 37.944; *p* < 0.001; partial η^2^ = 0.48; observed power = 1.00) showed that, overall, the percent of fails was smaller in the posttest (3.83% ± 4.54%) and retest (2.49% ± 2.90%) than in the pretest (7.41 ± 6.61 s; *p* < 0.001, for both), and in retest than in the posttest (*p* = 0.029). No other significant effects were observed.

### 3.2 Additional supporting experiment

A main effect of Time (*F*(2,28) = 15.888; *p* < 0.001; partial η^2^ = 0.53; observed power = 0.99) showed that response time was shorter in the posttest (426.44 ± 165.56 ms) and retest (441.35 ± 160.41 ms) than in the pretest (580.80 ± 113.52 ms; *p* = 0.02, for both). No other significant effects were observed (also for percent of fails).

## 4 Discussion

To the best of our knowledge, this is the first study to specifically assess whether ipsilateral transfer of a motor skill occurs from a unilateral UL to the LL, whereas previous studies have focused on transfer in the opposite direction (LL to UL) (Sherman et al., 2024) or bilateral limb transfer (Debarnot et al., 2024; Saeedpour-Parizi et al., 2022) or prism-induced visuomotor adaptation (Savin and Morton, 2008). We found that the response time of RM sequences of the LL was significantly faster (shorter) in the posttest in the group that practiced the RM sequence with the UL (UL group) compared to the group that observed nature movies (NO group), whereas it did not differ in the pretest between these groups. In addition, whereas in each group, response time improved significantly in the posttest and retest compared to the pretest, only in the NO group did response time also improve significantly in the retest compared to the posttest. The percent of fails improved (decreased) from pretest to posttest and retest and from posttest to retest, similarly in all groups.

Our finding that response time of RM sequences of the LL was significantly faster in the posttest in the UL group compared to the NO group is in line with our hypothesis that UL practicing would improve performance of LL compared to merely observing nature movies. This finding regarding ipsilateral transfer of performance from UL to LL supports the few previous studies that investigated unilateral ipsilateral transfer in healthy adults and youth (Magdi et al., 2021; Ben Othman et al., 2018). However, these studies focused on ipsilateral transfer of strength rather than motor skill, and various research has provided behavioral and neural evidence highlighting a distinction between strength and motor skill (Krakauer and Carmichael, 2022; Xu et al., 2017; Davidson and Buford, 2004). In addition, the previously investigated transfer was primarily from the LL to the UL. Our findings complement the recent study by Debarnot et al. (2024) and Saeedpour-Parizi et al. (2022) which also reported ipsilateral transfer of motor skills from the UL to the LL. However, whereas their research focused on a bilateral sequential motor learning task, our study examined a unilateral task. Interestingly, Debarnot et al. (2024) found that greater early transfer gains in performance were observed from the LLs to the ULs, which vanished after practice, and smaller early transfer benefits from the ULs to the LLs were observed, but they were durable. We did not directly compare ipsilateral transfer from the UL to the LL with ipsilateral transfer from the LL to the UL. However, in our recent study (Sherman et al., 2024), which examined the opposite transfer (LL to UL) using a similar setup to the current study, we found that reaching response time of the UL in the group practicing RM with the LL improved more than in the SO and NO groups in the posttest–an effect not observed in the current study (where the effect was greater compared to the NO group only). This suggests that LL practice enhanced UL performance more effectively than mere exposure to the cognitive aspect of the task (Sherman et al., 2024), whereas UL practice in the current study did not show the same effect on LL. Similar to the findings of Debarnot et al. (2024), our studies indicate that early ipsilateral transfer gains from the LL to the UL were more pronounced during the immediate posttest than the transfer from the UL to the LL. It should be noted, however, that in our previous study investigating ipsilateral transfer from the LL to the UL (Sherman et al., 2024), the number of RMs practiced by the ULs was greater than the number of movements practiced by the LLs in the current study (480 vs. 300 RMs).

The part of our hypothesis predicting faster posttest response time of RM sequences of the LL for the UL group compared to the SO group was not supported by our results. This hypothesis was based, as mentioned above, on Sherman et al.’s (2024) findings, where ipsilateral transfer from the LL to the UL showed improved posttest response time of RM sequences of the UL for the LL group over the SO group. The difference in practiced blocks between studies may explain why the current UL group did not differ from the SO group. In the current study, the UL group practiced 16 blocks, while the LL group in Sherman et al. (2024) practiced only 10 blocks, possibly reducing task specificity and increasing transfer effects in the latter study. Alternatively, a larger dose practice of LL RM practice (than the one used in the current study) could have strengthened the motor component of learning and thereby increase the difference between the UL and SO groups. In the current study, the coefficient of variation (CV; SD/mean) increased from pretest to posttest across all groups (SO: pretest: 14.46%, posttest: 28.78%; NO: pretest: 11.4%, posttest: 15.4%), with the largest rise in the UL group (pretest: 9.08%, posttest: 33.22%). Because decreasing variability is typically considered a marker of the onset of learning (Adi-Japha et al., 2008), this elevated CV may reflect an early stage of ipsilateral transfer (possibly leading to non-significant difference between UL and SO groups). Overall, under the present protocol, ipsilateral transfer appears to reflect contributions from both cognitive (effector-independent sequence knowledge) and motor processes. Future work that increases repetitions within a session and/or introduces multiple sessions will help determine whether motor practice can surpass cognitive exposure in driving ipsilateral transfer of sequential motor task.

In our study, the response time of RM sequences of the LL improved in the UL group in the posttest and retest compared to the pretest following the practice of 16 blocks of RM sequences with the UL, but did not further improve from the posttest to the retest, i.e., there was no off-line consolidation of the ipsilateral transfer of the LL. Interestingly, based on the additional supporting experiment that we conducted, we found that the subjects’ ability to perform RM sequences with the UL following the practice of 16 blocks of RM sequences with the UL in an identical setup also improved in the posttest and retest compared to the pretest, but without further improvement from posttest to retest. It seems that the structure of the UL practice session, which included 16 blocks (consisting of 480 RMs), was insufficient to trigger off-line gains in the UL performance. The fact that the task of RM sequences with the UL did not fully consolidate and became specific, even following the practice of 480 RMs, probably enabled the ipsilateral transfer from UL to the LL in the UL group (Hauptmann et al., 2005).

Our data align with the generalized motor program theory, which views motor learning as the development of an abstract memory structure (a motor program) that allows for adaptation of learned skills to changing environments (Schmidt and Young, 1987). This central motor representation is thought to be independent of the effector used, supporting inter- and intramanual transfer. Ipsilateral transfer may involve the rolandic motor association (RMA) region within the central sulcus, a motor association area, shown by intracranial sEEG signals to be active during movements of the tongue, hands, and feet (Jensen et al., 2023). Its activity across different effectors suggests that the RMA may serve as a shared representation area, coordinating movements between body parts, thus helping to integrate and manage motor outputs across various effectors. Complementary evidence from precision functional magnetic resonance imaging (fMRI) methods indicates the classic homunculus is interrupted by regions with distinct connectivity (with one another and with the cingulo-opercular network), structure and function that alternate with effector-specific (foot, hand and mouth) areas (Gordon et al., 2023). The inter-effector regions lacked movement specificity and co-activated during action planning (coordination of hands and feet) and axial body movement. In that study, the authors suggested that M1 is comprising two intertwined systems–effector-specific regions that support fine, isolated control, and a somato-cognitive action network that integrates goals, physiology, and body movement.

Our RM sequence task involves both motor and cognitive aspects. The cognitive aspect relates to the repeated exposure to the illuminating switches, as each illuminated LED cue prompted subjects to reach and press the push-button switch. Although participants were not explicitly informed about the sequence, repeated exposure during the session (80 sequences for both UL and SO groups) likely led to familiarization, allowing them to memorize the sequence. This cognitive familiarity may have contributed to the improved response times for the LL. However, the lack of significant posttest improvement in the SO group compared to the NO group suggests that cognitive practice alone was insufficient to trigger ipsilateral transfer from the UL to the LL. With respect to the motor aspect, we hypothesized that practicing RM sequences with the UL would improve LL performance more than simply observing the same sequences, due to the active motor practice. The UL group was instructed to reach and press the illuminated switch as quickly and accurately as possible, while the SO group only observed the sequence without moving. By comparing these groups, we aimed to isolate the motor and cognitive aspects. However, the finding that the response time of the RM sequences of the LL in the UL group was not faster than in the SO group suggests that motor practice alone was not superior to cognitive exposure in producing ipsilateral transfer. These results, along with the faster response times for the LL RM sequences in the UL group compared to the NO group, suggest that a combination of motor and cognitive practice was required to induce ipsilateral transfer of the RM sequence from the UL to the LL. This effect was likely facilitated by the development of an effector-independent motor representation (Schmidt and Young, 1987; Schmidt, 1975) and the sharing of a cognitive strategy.

The role of cognitive strategy in intermanual transfer remains debated. Some studies indicate that explicit cognitive processes can enhance intermanual transfer (Bouchard and Cressman, 2021; Malfait and Ostry, 2004; Werner et al., 2004; De Havas et al., 2022), particularly when participants are aware of large visuomotor distortions. Similarly, in tasks with endpoint feedback, where participants applied isometric force to adjust the height of a visual bar to a target level, intermanual transfer was facilitated when only endpoint feedback was provided. This condition relied more on cognitive strategies, as shown by increased reaction times, suggesting that effector-independent learning was supported by cognitive strategy (De Havas et al., 2022). However, other evidence suggests that awareness of visuomotor perturbations is not always necessary for intermanual transfer. For example, informing participants about a visuomotor rotation before adaptation did not improve transfer compared to conditions without prior explanation (Wang et al., 2011). Additionally, small perturbations (22.5° or 32°), whether introduced abruptly or gradually, showed no significant differences in transfer outcomes (Taylor et al., 2011), possibly because the perturbations were too small to elicit sufficient awareness. Various factors, such as perturbation size (Taylor et al., 2011; Werner et al., 2015), which hand is trained first, and the spatial location of targets, also affect the extent of intermanual transfer (Mostafa et al., 2014; Wang and Sainburg, 2006). These findings suggest that cognitive strategies can influence intermanual transfer, but their impact may vary depending on specific task conditions.

Exposure solely to the cognitive aspect of the task (SO group) did not result in ipsilateral transfer. However, it influenced retest performance, similar to the UL group, as the RM sequences of the LL improved in the retest compared to the posttest only in the NO group. The improvement in the LL response time following 24 h in the NO group can be related to a slow, across-session learning phase due to an off-line slow evolving consolidation (Karni et al., 1998; Karni, 1997). The number of RM sequences repetitions practiced by the LL during the pretest and posttest (a total of 180 repetitions) was enough for consolidation of LL response time in the retest. Interestingly, in a similar study (Sherman et al., 2024) examining the opposite ipsilateral transfer from the LL to the UL, the NO group did not show improvement in the UL RM sequence response time from posttest to retest. This may be attributed to the lower number of RM sequence repetitions practiced by the UL during the pretest and posttest (a total of 120 repetitions) compared to the 180 repetitions in the current study. However, factors beyond practice volume likely shaped consolidation in the current study, given that UL and SO groups (who completed the same amount of RM sequence repetitions in the tests as the No group) did not improve from posttest to retest. Exposure only to the sequence itself, with and even without UL practice (UL and SO groups, respectively), may have interfered with consolidation processes of the LL sequence performance. Alternatively, the additional reduction in LL response time in the NO group could be attributed to the slower performance of this group in the posttest compared to the UL and SO groups, potentially allowing more room for improvement in the retest (however, the LL response time in the NO group was significantly slower only in comparison to the UL group in the posttest). A further explanation may relate to activities and sleep undertaken during the 24-h interval between posttest and retest in each group (Korman et al., 2003, 2007; Dudai et al., 2015). Nocturnal sleep duration and quality did not differ between groups. However, we did not monitor participants’ activities and additional sleep during this period. Future studies should track intersession sleep and activity to ensure these factors do not confound between-group differences.

### 4.1 Limitations of study

The study has several limitations. First, given the potential influence of baseline individual cognitive function (e.g., attention, memory, executive function) on task performance and motor learning (Song, 2019; Bao et al., 2024), adding a brief cognitive screening would help ensure that differences in general cognitive abilities do not confound the results. Second, adding a post experiment questionnaire to assess the subjects’ cognitive awareness (for a similar approach, see Bouchard and Cressman, 2021) of the sequences could have clarified whether the ipsilateral transfer was also related to explicit knowledge of the sequence due to memorizing it. Third, separately measuring reaction time and movement time could have provided greater focus on ipsilateral transfer of motor performance, which is primarily reflected in movement time. Alternatively, examining a randomized sequence would allow for an assessment of motor reaching skill improvement, independent of sequence-specific learning. Fourth, the experimenter was aware of the group allocation. However, it is important to highlight that the motor task scoring was performed automatically by the LabVIEW software, minimizing the potential for bias. Finally, our study comprised young, healthy adults; future work should examine ipsilateral transfer in older adults and clinical populations (e.g., stroke) to enhance translational relevance.

## 5 Conclusion

Our results provide evidence for ipsilateral transfer of a motor skill from the UL to the LL among healthy adults. From a mechanistic perspective, future studies should incorporate cognitive assessments to more precisely determine the role of cognitive abilities in ipsilateral skill transfer. From a practical perspective, the potential of ipsilateral transfer as a strategy for enhancing motor skills in both healthy individuals, such as athletes, and patients with movement disorders should be further explored. In conditions where an individual’s LL is impaired and direct training is not feasible, training the UL may contribute to improving the motor abilities of the LL.

## Data Availability

The original contributions presented in this study are included in this article/Supplementary material, further inquiries can be directed to the corresponding author.

## Supplementary material

Supplementary Table 1 Individual data in time points. Ms, milliseconds. LL group, lower limb group which practiced reaching movements sequence with the LL toward light switches; SO group, switches observation group which observed the sequence of light switches; NO group, nature observation group which observed nature films; F, female; M, male.
