# Supplementary material for: Ipsilateral transfer of motor skill from upper to lower limb in healthy adults: a randomized controlled trial
Source: Front Hum Neurosci. 2025 Nov 4;19:1645986. doi: 10.3389/fnhum.2025.1645986 (PMC12623383; doi:10.3389/fnhum.2025.1645986)
Supplement: Supplementary Table 1 — Individual data in time points. Ms, milliseconds. LL group, lower limb group which practiced reaching movements sequence with the LL toward light switches; SO group, switches observation group which observed the sequence of light switches; NO group, nature observation group which observed nature films; F, female; M, male. [file Table_1.docx]

**Title:** **Individual data in time points**

| Subject code | Group | Sex | Age | Pretest-Response time (ms) | Posttest-Response time (ms) | Retest-Response time (ms) | Pretest-Fails (%) | Posttest-Fails (%) | Retest-Fails (%) |
| --- | --- | --- | --- | --- | --- | --- | --- | --- | --- |
| 1 | SO | M | 26 | 648.5 | 607.5 | 449.6 | 3.3 | 2.2 | 3.3 |
| 2 | NO | F | 27 | 670.5 | 625.0 | 600.5 | 11.1 | 6.7 | 1.1 |
| 3 | NO | F | 24 | 639.2 | 625.6 | 543.1 | 6.7 | 3.3 | 1.1 |
| 4 | NO | M | 27 | 831.5 | 787.8 | 695.5 | 36.7 | 26.7 | 15.6 |
| 5 | UL | F | 28 | 773.2 | 533.4 | 531.7 | 16.7 | 12.2 | 7.8 |
| 6 | UL | F | 22 | 650.7 | 698.8 | 657.0 | 1.1 | 1.1 | 1.1 |
| 7 | NO | M | 27 | 617.3 | 520.7 | 423.7 | 13.3 | 7.8 | 6.7 |
| 8 | SO | M | 29 | 776.8 | 669.5 | 687.1 | 6.7 | 2.2 | 3.3 |
| 9 | UL | F | 25 | 681.3 | 374.4 | 515.9 | 13.3 | 4.4 | 4.4 |
| 10 | SO | F | 24 | 803.0 | 601.9 | 480.6 | 20.0 | 3.3 | 2.2 |
| 11 | SO | F | 26 | 551.0 | 313.5 | 244.8 | 3.3 | 1.1 | 0.0 |
| 12 | SO | F | 33 | 628.3 | 575.6 | 603.4 | 5.6 | 1.1 | 4.4 |
| 13 | UL | F | 24 | 721.8 | 534.2 | 603.9 | 6.7 | 0.0 | 7.8 |
| 14 | NO | F | 24 | 650.0 | 518.4 | 600.5 | 2.2 | 3.3 | 1.1 |
| 15 | UL | M | 26 | 733.8 | 639.2 | 643.0 | 5.6 | 2.2 | 1.1 |
| 16 | SO | F | 24 | 698.1 | 394.8 | 338.3 | 16.7 | 1.1 | 1.1 |
| 17 | UL | F | 25 | 604.1 | 602.4 | 543.1 | 5.6 | 7.8 | 1.1 |
| 18 | UL | M | 26 | 540.7 | 285.4 | 233.2 | 0.0 | 0.0 | 1.1 |
| 19 | SO | M | 26 | 615.7 | 300.9 | 265.5 | 2.2 | 3.3 | 1.1 |
| 20 | UL | F | 23 | 657.3 | 411.3 | 385.7 | 11.1 | 1.1 | 2.2 |
| 21 | SO | F | 21 | 759.6 | 606.1 | 515.4 | 13.3 | 1.1 | 1.1 |
| 22 | UL | M | 26 | 628.4 | 281.4 | 246.6 | 4.4 | 1.1 | 0.0 |
| 23 | UL | M | 26 | 623.8 | 524.2 | 544.5 | 8.9 | 8.9 | 4.4 |
| 24 | NO | M | 28 | 553.8 | 522.7 | 399.1 | 4.4 | 2.2 | 0.0 |
| 25 | NO | M | 27 | 546.3 | 500.1 | 437.0 | 6.7 | 8.9 | 3.3 |
| 26 | NO | M | 26 | 646.8 | 654.7 | 605.0 | 5.6 | 2.2 | 4.4 |
| 27 | UL | F | 24 | 622.1 | 344.7 | 271.1 | 5.6 | 3.3 | 0.0 |
| 28 | NO | F | 21 | 709.6 | 698.2 | 660.6 | 6.7 | 7.8 | 4.4 |
| 29 | UL | F | 26 | 693.9 | 358.2 | 345.3 | 8.9 | 2.2 | 1.1 |
| 30 | SO | F | 26 | 735.3 | 708.8 | 684.0 | 16.7 | 6.7 | 3.3 |
| 31 | NO | F | 26 | 593.3 | 566.3 | 549.8 | 2.2 | 3.3 | 5.6 |
| 32 | SO | M | 28 | 551.7 | 599.2 | 550.3 | 1.1 | 0.0 | 2.2 |
| 33 | SO | F | 25 | 601.8 | 413.1 | 411.1 | 2.2 | 2.2 | 0.0 |
| 34 | UL | M | 25 | 617.8 | 326.8 | 319.5 | 4.4 | 1.1 | 0.0 |
| 35 | NO | F | 24 | 622.2 | 593.2 | 539.5 | 2.2 | 2.2 | 0.0 |
| 36 | NO | F | 24 | 605.4 | 578.4 | 550.3 | 10.0 | 5.6 | 3.3 |
| 37 | SO | M | 26 | 601.4 | 425.4 | 341.7 | 7.8 | 3.3 | 2.2 |
| 38 | NO | F | 25 | 537.5 | 415.6 | 279.7 | 3.3 | 2.2 | 0.0 |
| 39 | SO | M | 26 | 785.4 | 516.3 | 463.1 | 10.0 | 2.2 | 4.4 |
| 40 | NO | M | 28 | 632.4 | 541.3 | 512.6 | 2.2 | 2.2 | 1.1 |
| 41 | NO | M | 25 | 649.7 | 590.2 | 537.7 | 1.1 | 1.1 | 1.1 |
| 42 | UL | M | 27 | 638.4 | 289.4 | 358.6 | 3.3 | 2.2 | 1.1 |
| 43 | UL | M | 29 | 607.0 | 277.8 | 300.6 | 1.1 | 0.0 | 0.0 |
| 44 | SO | M | 27 | 509.8 | 238.7 | 357.6 | 4.4 | 0.0 | 0.0 |
| 45 | SO | M | 26 | 598.9 | 572.2 | 516.2 | 8.9 | 8.9 | 1.1 |
